# LDHA Lactylation Drives Malignant Progression of Oral Squamous Cell Carcinoma via a Positive Feedback Loop with Lactate

**DOI:** 10.7150/ijbs.136949

**Published:** 2026-09-10

**Authors:** Fan Song, Chen Hou, Yingzhao Huang, Hongshi Cai, Jianfeng Liang, Yaoqi Jiang, Qiong Xu, Jinsong Hou

**Affiliations:** 1 Hospital of Stomatology, Guanghua School of Stomatology, Sun Yat-Sen University, Guangzhou, Guangdong 510055, China.; 2 Guangdong Provincial Key Laboratory of Stomatology, Sun Yat-Sen University, Guangzhou, Guangdong 510055, China.

**Keywords:** oral squamous cell carcinoma, lysine lactylation, LDHA, lactate, glycolysis, GCN5

## Abstract

Lysine lactylation (Kla) is an emerging post-translational modification closely linked to glycolysis, where it functions to regulate the activity of glycolytic enzymes. In oral squamous cell carcinoma (OSCC), elevated expression of Lactate dehydrogenase A (LDHA) drives lactate production, thereby supplying the substrate for Kla. Nevertheless, whether LDHA itself undergoes Kla in OSCC has yet to be explored. Here, we have conclusively verified that LDHA undergoes high-level Kla in OSCC tissues and cells, with its Kla abundance positively associated with intracellular lactate concentrations. Both *in vitro* and *in vivo* analyses collectively illustrated that LDHA-K318 lactylation functions as a crucial driver of OSCC malignant progression. Functionally, our investigations revealed that the LDHA-K318R mutation diminishes lactate generation and decreases global protein Kla levels. GCN5 acts as an upstream "writer" that coordinately regulates LDHA-Kla, thereby modulating lactate production and facilitating OSCC malignancy. Furthermore, we identified HDAC1 as an "eraser" that governs LDHA-Kla status. Combined administration of the GCN5 inhibitor MB-3 and the LDHA-K318 lactylation-targeting peptide (K318-pe) remarkably inhibited tumor growth in the PDX models. Our study uncovers a positive feedback loop linking lactate and glycolysis, mediated by LDHA-Kla in OSCC. This discovery implicates lactylation-associated regulators as viable therapeutic targets, opening novel perspectives for clinical application.

## Introduction

Oral squamous cell carcinoma (OSCC) represents the most prevalent malignancy in the oral and maxillofacial region, with recurrence and metastasis being the primary causes of mortality among patients^1^, ^2^. Elucidating the mechanisms that govern invasion and metastasis is therefore critical for the development of effective therapeutic strategies. Aerobic glycolysis, widely recognized as the Warburg effect, constitutes the predominant metabolic phenotype of tumors^3^-^5^. Emerging evidence has demonstrated that lactate, the end product of aerobic glycolysis, serves not only as an energy source and biosynthetic precursor for tumor cells but also as a critical substrate that drives protein lysine L-lactylation (Kla)^6^-^8^. Lactylation is critically involved in various oncogenic processes encompassing epigenetic dysregulation, angiogenesis, DNA damage repair, autophagy, immune evasion, and chemoresistance, which together contribute to tumor development, progression, invasion, and metastasis^9^-^13^. Notably, the Warburg effect is a core feature of metabolic reprogramming in OSCC^14^. Lactate can facilitate lactylation on both histones and non-histone proteins in OSCC^15^. Nevertheless, the precise biological functions and regulatory mechanisms underlying protein lactylation in OSCC progression remain largely unexplored.

Lactate dehydrogenase A (LDHA), a pivotal enzyme governing lactate generation in the glycolytic pathway, efficiently catalyzes the conversion of pyruvate to lactate, accompanied by the release of energy, NAD+, and H_2_O. Previous studies have confirmed that LDHA is highly expressed in various malignancies, including OSCC, where it functions as a positive regulator driving tumor progression^16^-^18^. The expression and activity of LDHA are subject to multifaceted regulation at the transcriptional level, epigenetic modification, and post-translational modification (PTM)^19^. Various PTMs, such as phosphorylation, acetylation, and methylation, modulate LDHA enzymatic activity and protein stability, thereby influencing diverse physiological and pathological processes^20^-^23^. Our preliminary findings have revealed that a large number of proteins in OSCC notably undergo lactylation, including several metabolic enzymes within the glycolytic pathway. Moreover, the data suggest the presence of two potential lactylation sites on LDHA^15^. However, whether LDHA is lactylated in OSCC and how such lactylation modulates malignant phenotypes remain to be elucidated.

L-lactylation is an enzymatic modification that is dynamically regulated by the coordinated action of "writers" and "erasers," which govern the addition and removal of this modification on substrates^24^. To date, several acetyltransferases and alanyl-tRNA synthetase—including P300, CBP, GCN5, TIP60, MOF and AARS1—have been identified as capable of catalyzing lactylation, whereas HDAC1/2/3 and SIRT1/2/3 serve as the primary delactylases^10^, ^24^-^31^. Building on our confirmation of LDHA lactylation in OSCC, we aim to identify the specific writer and eraser responsible for regulating this modification and to explore its functional contribution to malignant progression of OSCC.

## Materials and Methods

### Antibodies and reagents

**Antibodies for western blotting**: Pan Kla (1:1000, PTM-1401RM, PTM BIO, China, RRID: AB_2942013), LDHA (1:5000, 19987-1-AP, Proteintech, China), HA (1:5000, 51064-2-AP, Proteintech, China), CBP (1:1000, D6C5, CST, USA), P300 (1:1000, D8Z4E, CST, USA), GCN5 (1:1000, ER63516, HUABIO, China), AARS1 (1:10000, 87581-2-RR, Proteintech, China), PCAF (1:5000, 86917-1-RR, Proteintech, China), MOF (1:1000, 13842-1-AP, Proteintech, China), TIP60 (1:1000, 10827-1-AP, Proteintech, China), HDAC1 (1:10000 ,83624-1-RR, Proteintech, China), HDAC2 (1:10000, 67165-1-Ig, Proteintech, China), HDAC3 (1:10000, 81211-1-RR, Proteintech, China), HRP Conjugated GAPDH (1:100000, HA724237H, HUABIO, China), HRP Conjugated β-actin (1:50000, HA724235H, HUABIO, China), HRP Conjugated β-tubulin (1:10000, HA724236H, HUABIO, China), HRP Goat Anti-Rabbit IgG secondary antibody (1:4000, 7074, CST, USA) and HRP-conjugated Goat Anti-Mouse secondary antibody (1:4000, SA00001-1, Proteintech, China), IPKine™ HRP, Mouse Anti-Rabbit IgG LCS (1:2000, A25022, Abbkine, China).

**Antibodies for immunoprecipitation/ co-immunoprecipitation**: Pan Kla (1:200, PTM-1401RM, PTM BIO, China), LDHA (4.0 ug for 3.0 mg of total protein lysate, 19987-1-AP, Proteintech, China), HA (4.0 ug for 3.0 mg of total protein lysate, 51064-2-AP, Proteintech, China), Normal Rabbit IgG (1:200, 2729S, CST, USA).

**Antibodies for immunofluorescence staining/ immunohistochemistry:** Pan Kla (1:200, PTM-1401RM, PTM BIO, China), GCN5 (1:100, ER63516, HUABIO, China), GCN5 (1:400, 66575-1-Ig, Proteintech, China), LDHA (1:200, 19987-1-AP, Proteintech, China), Ki67 (1:200, ab16667, Abcam, USA), Alexa Fluor594, Goat Anti-Rabbit IgG(H+L) (EM35153-01, 1:200, EMAR, China).

**Reagents**: L-Sodium Lactate (Nala, S108838, Aladdin, China), 2-Deoxy-D-glucose (2DG, D109194, Aladdin, China), Panobinostat (S1030, Selleck, USA), Nicotinamide (NAM, HY-B0150, MCE, USA), Butyrolactone 3 (MB-3, HY-129039, MCE, USA), HA Peptide (HY-P0239, MCE, USA), K318-peptide (Guoping Pharmaceutical, China, detailed information in Table S1), DAPI (C1006, Beyotime, China).

### OSCC samples

A total of 71 OSCC and 20 adjacent noncancerous tissue specimens were retrospectively collected from patients treated at the Affiliated Stomatology Hospital of Sun Yat-sen University between 2021 and 2022. Human OSCC tissues were collected for the establishment of PDX models. Written informed consent was obtained from all participants prior to enrollment, and the study protocol was approved by the Institutional Research Ethics Committee of the same institution (approval No. KQEC-2020-16-02, KQEC-2020-16-03, KQEC-2025-192-01).

### Cell culture

The human OSCC cell lines SCC25, HSC3, CAL233, and HN6, along with human embryonic kidney-derived 293T (293T) cells and the normal oral squamous epithelial cell line NOK, were originally procured from the American Type Culture Collection (ATCC). SCC25 and HN6 cells were maintained in DMEM/Ham's F12 medium (DMEM/F12, Gibco, USA) supplemented with 10% FBS. HSC3, CAL33 and 293T cells were cultured in DMEM medium (Gibco, USA) containing 10% FBS. For normoxic conditions, cells were cultured at 37 °C in a humidified atmosphere containing 21% O₂, 5% CO₂, and 74% N₂. Hypoxic exposure was conducted in a hypoxia humidified incubator (ThermoFisher, USA) with 1% O₂, 5% CO₂, and 94% N₂ for the indicated durations. All cell manipulations were performed within a sterile biosafety cabinet (ThermoFisher, USA).

### Proteomic quantification of lysine lactylation

OSCC cells cultured under normoxic and hypoxic conditions were collected and sent to PTM Biolabs (Hangzhou, China) for liquid chromatography-tandem mass spectrometry (LC-MS/MS) quantitative analysis of the lysine lactylome. The experimental workflow included TMT labeling, high-performance liquid chromatography (HPLC) fractionation, affinity enrichment, and mass spectrometry-based quantitative proteomics. Subsequent bioinformatic analysis involved functional annotation, classification, enrichment, and cluster analysis of quantified lactylated proteins. The detailed experimental and analytical methods are described in this article^15^.

### Western blotting (WB)

Cells were lysed in RIPA buffer (CW2333S, CWBIO, China) supplemented with a protease inhibitor cocktail (CW2200S, CWBIO, China), followed by centrifugation at 14,000g for 20 min at 4 °C. Protein concentrations were determined using a BCA kit (CW0014S, CWBIO, China) according to the manufacturer's instructions. Protein samples were resolved by 10% SDS-PAGE (PG112, Epizyme, China) and transferred onto PVDF membranes (ISEQ00010, Millipore, USA). Following blocking with 5% skim milk (232100, BD, USA) for 2h at room temperature, the membranes were incubated overnight at 4°C with the indicated primary antibodies. After washing for three times, the membranes were incubated with HRP-conjugated secondary antibody for 1h at room temperature. Lastly, the blots were developed using enhanced chemiluminescence (ECL) substrate (WBKLS0500, Millipore, USA).

### Lentiviral transfection and transient transfection

Stable cell lines were generated via a lentiviral delivery system. Two shRNA sequences targeting LDHA were individually inserted into the pLKO.1-puro lentiviral vector (Jiangsu Saisofi Biotechnology Co., Ltd, China), with detailed sequences provided in Table S2. To achieve LDHA overexpression, the full-length cDNA was subcloned into the pLVoeRNA-G418 lentiviral vector, whereas constructs expressing LDHA-WT, LDHA-K81R, and LDHA-K318R (Jiangsu Saisofi Biotechnology Co., Ltd, China) (Table S3). Lentiviral particles were produced by co-transfecting 293T cells with the relevant target plasmids along with psPAX2 and pMD2.G using Lipofectamine 8000 (C0533, Beyotime, China) for 6 hours, and supernatants were collected 48-72 hours post-transfection. HSC3 and SCC25 cells seeded in 6-well plates were transduced with viral particles in the presence of 10 μg/mL polybrene (H8761, Solarbio, China), followed by puromycin or G418 selection for two weeks. siRNAs targeting HDAC1/2/3, siGCN5 and a siNC were synthesized by Jidan (Guangzhou, China) based on the sequences provided in Table S4 and Table S5. Transient knockdown was achieved by transfecting the siRNAs using Lipofectamine 8000 reagent (C0533, Beyotime, China) for 3-4 days following the manufacturer's instructions. Knockdown and overexpression efficiencies were assessed by western blotting.

### Cell proliferation and colony formation assay

Cells at the logarithmic growth phase were routinely digested, centrifuged, and the supernatant was discarded. After cell counting, appropriate numbers of cells were seeded into 96-well plates (SCC25: 3000 cells/100 μL/well; HSC3: 2000 cells/100 μL/well). Cell proliferation was assessed using the Cell Counting Kit-8 (40203ES80, Yeasen, China), following the manufacturer's instructions and our previous protocol^32^. In the colony formation assay, HSC3 and SCC25 were plated in 12-well plates at densities of 800 and 1000 cells per well, respectively. Following a 10-14 days incubation period, the cells were fixed with 4% paraformaldehyde for 30 minutes and subsequently stained with 0.1% crystal violet for 20 minutes.

### Cell migration and invasion assay

Cell migration and invasion were assessed following the protocol detailed in our prior study (SCC25: 8**×**10^4^ cells/200 μL/upper chambers; HSC3: 5**×**10^4^ cells/200 μL/upper chambers)^32^. Images were obtained from five randomly fields per chamber using an inverted microscope (Zeiss Axio, Zeiss, Germany).

### Immunoprecipitation (IP) and co-immunoprecipitation (Co-IP)

Cells were lysed in Co-IP lysis buffer (PR20037, Proteintech, China) supplemented with a protease inhibitor cocktail (CW2200S, CWBIO, China), followed by centrifugation at 14,000 g for 20 min at 4 °C. Protein concentration was determined using a BCA protein assay kit (CW0014S, CWBIO, China) following the manufacturer's instructions. For IP, supernatants containing equal amounts of total protein were incubated with the indicated primary antibodies overnight at 4 °C to allow formation of antigen-antibody complexes. These complexes were then incubated with Protein A/G magnetic beads (HY-K0202, MCE, USA) for 2-4h at 4°C to generate bead-antigen-antibody complexes, after which the beads were captured using a magnetic separator and the supernatant discarded. For Co-IP, equal amounts of protein lysates were directly incubated with Anti-HA Magnetic Beads (HY-K0201, MCE, USA) overnight at 4 °C to capture HA-tagged proteins, followed by magnetic separation and supernatant removal. The bead-bound immunocomplexes were washed three times with wash buffer (0.5% PBST) before denaturation and elution. Finally, the prepared samples were subjected to western blotting analysis as described previously.

### Intracellular lactate assay

Cells were harvested either with or without the indicated treatments and processed accordingly. Samples were lysed on ice using a high-intensity ultrasonic processor (Scientz) for 5 minutes under the following conditions: 200 W output power, 3-second pulses interspersed with 7-second intervals, repeated 20 times. The lysates were then centrifuged at 12,000 × g for 5 minutes at 4°C to isolate the supernatant. Lactate levels in the supernatant were subsequently quantified using the CheKine™ Lactate Assay Kit (KTB1100, Abbkine, China) in accordance with the manufacturer's protocol.

### Nuclear and cytosolic fractionation assay

Separation of nuclear and cytoplasmic protein extracts was carried out using the Nuclear and Cytoplasmic Protein Extraction Kit (P0027, Beyotime, China) strictly according to the manufacturer's instructions. Each fraction was then resolved by SDS-PAGE and subjected to western blot analysis.

### Protein stability assay

Protein stability of LDHA was assessed by cycloheximide chase assay. OSCC cells were treated with cycloheximide (CHX, 50 uM) to inhibit new protein synthesis. Cell lysates were harvested at the indicated time points (0, 2, 4, 8, 10, 12h) and subjected to western blot analysis. The protein band intensity was quantified and normalized to the loading control.

### Measurement of LDHA Enzyme Activity

HA-LDHA-WT, HA-LDHA-K81R, and HA-LDHA-K318R were individually introduced into HEK293T cells, followed by immunoprecipitation using HA magnetic beads. The bound proteins were eluted in buffer [20 mM Hepes, 150 mM NaCl, 1 mM EDTA, 3×HA peptide (200μg·mL⁻¹), pH 7.5], and purified LDHA proteins (1μg) were subsequently assayed in reaction buffer [0.2M Tris-HCl, 30mM pyruvate, 2 mM NADH, pH 7.4]. Absorbance readings at 340nm were recorded spectrophotometrically.

### Immunofluorescence staining (IF)

OSCC cells (1×10⁴) were seeded into confocal dishes and cultured under the indicated conditions for the specified durations. After treatment, cells were fixed with 4% paraformaldehyde for 20 min, rinsed three times with PBS (5 min each wash). After permeabilization with 0.1% Triton X-100 for 10 min, cells were blocked with 5% bovine serum albumin (BSA) at room temperature for 1h, followed by three additional PBS washes (5 min each). Subsequently, the samples were incubated overnight at 4°C with primary antibodies. After washing, Alexa Fluor 594-conjugated goat anti-rabbit IgG (H+L) and Alexa Fluor 488-conjugated goat anti-mouse IgG (H+L) were applied for 1 h at room temperature, and nuclei were counterstained with DAPI. Images were acquired using a confocal microscope (LSM 980, Zeiss, Germany).

### *In vivo* experiments

#### Tumor xenografts

In the tumor formation assay, 8-10-week-old male NOD/SCID mice (Sun Yat Sen University, Guangzhou, China) were randomly allocated into four groups: shLDHA+LDHA-WT+DMSO, shLDHA+LDHA-WT +MB-3, shLDHA+LDHA-K318R+DMSO, shLDHA+LDHA-K318R+MB-3 (n = 6 per group). After intraperitoneal injection of 2% pentobarbital sodium for anesthesia, approximately 2×10^7^ SCC25 cells resuspended in 150 µL of DMEM/Matrigel mixture (volume =2:1) were injected subcutaneously into the left axilla of mice to establish a tumor xenograft. One week after tumor inoculation, body weight and tumor volume were measured every 3 days until mice exhibited a weight loss of more than 15% within a short period or were euthanized. Tumor volume (V) was calculated using the formula: V = 0.5 × length × width². Three weeks after cell implantation, mice in the MB-3-treated groups received intraperitoneal injection of MB-3 at a dose of 5 mg/kg every other day. After two weeks of treatment, subcutaneous xenograft tumors were harvested for embedding, sectioning, and staining. All animal procedures were approved by the Animal Ethics Committee of Sun Yat-sen University (SYSU-IACUC-2024-001081). The mice were sacrificed and the tumors were harvested to compare tumor burdens.

#### Patient-derived xenograft models (PDX)

For PDX model establishment, freshly resected tumor specimens were immediately placed on ice and transferred to the laboratory within 1h in DMEM supplemented with 50 U·ml⁻¹ penicillin-streptomycin. After triple washing with ice-cold antibiotic-containing medium, tumor tissues were minced into small fragments using sterile surgical blades. The freshly prepared tissue fragments were subcutaneously implanted into the axillary fossa of anesthetized BALB/c Nude mice via minor surgical incisions. Incision sites were closed with sutures, and tumor development was monitored for the following 2 weeks. Tumor-bearing mice were randomly assigned to four treatment groups: PBS vehicle control, K318-pe (5 mg/kg, intraperitoneal injection once daily), MB-3 (5mg/kg, intraperitoneal injection once daily), and combined K318-pe and MB-3 (5 mg/kg each, intraperitoneal injection once daily). Following 10 consecutive days of treatment, all mice were humanely euthanized, and tumors were surgically dissected and processed for fixation. The demographic and clinical characteristics of OSCC patients contributing primary tumor tissue for PDX generation are detailed in Table S6. All animal procedures were approved by the Animal Ethics Committee of Sun Yat-sen University (SYSU-IACUC-2025-B0557).

#### Immunohistochemistry (IHC)

A total of 71 OSCC and 20 adjacent noncancerous tissue specimens were retrospectively collected from patients treated at the Affiliated Stomatology Hospital of Sun Yat-sen University between 2018 and 2022. Harvested OSCC and adjacent noncancerous tissues were immediately fixed in 4% paraformaldehyde, routinely dehydrated, embedded in paraffin, and serially sectioned into 4-μm thick slices for subsequent immunohistochemical (IHC) staining using a DAB Detection Kit (GK600710, GeneTech, Shanghai) following the manufacturer's instructions. Leica Aperio AT2 (Leica, Germany) was used for slices scanning.

#### Bioinformatics analysis

GCN5 (KAT2A) transcriptomic data were downloaded from The Cancer Genome Atlas (TCGA) for multiple cancer types, including head and neck squamous cell carcinoma, and were extracted and integrated using R software packages. Samples with complete GCN5 transcriptomic data were selected for further analysis. Statistical analyses were performed to evaluate the differential expression of GCN5 between multiple tumor tissues and their adjacent normal tissues. Additionally, information of OSCC samples was extracted to compare the differential expression of GCN5 between paired and unpaired cancer tissues and adjacent normal tissues.

### Statistical analysis

All statistical analyses were performed using GraphPad Prism 11. Comparisons between two groups were evaluated using two-tailed unpaired Student's t-tests, whereas comparisons involving multiple groups were assessed by one-way ANOVA. To analyze the relationship between a categorical variable and a quantitative variable, the Wilcoxon rank-sum test was performed. *P*-values lower than 0.05 were deemed statistically significant.

## Results

### 3.1 LDHA is hyperlactylated in OSCC

Previous research has mapped the Kla profile in OSCC by performing LC-MS/MS analysis on OSCC cells cultured under hypoxic and normoxic conditions. The results showed that multiple metabolic enzymes in the glycolysis pathway undergo Kla, among which LDHA, a key enzyme regulating lactate production, also contains Kla sites^15^ (Figure 1A). Based on our team's previous findings that LDHA is highly expressed in OSCC and promotes malignant progression of OSCC, this study aims to explore the effect of LDHA-Kla on the biological behavior of OSCC^16^. Firstly, to verify the presence of Kla on LDHA in OSCC, proteins were prepared from 5 paired of OSCC and adjacent non-tumor tissues. We demonstrated that LDHA was highly expressed in OSCC tissues and exhibited pronounced Kla modification via IP and WB analyses (Figure 1B). Similarly, significant LDHA-Kla was observed in OSCC cell lines SCC25 and HSC3 (Figure 1C). Treatment with Nala induced a dose-dependent increase in LDHA Kla levels, while treatment with the glycolysis inhibitor 2-deoxy-D-glucose (2DG) showed the opposite trend (Figure 1D-E). In addition, hypoxic culture conditions markedly enhanced the Kla modification of LDHA in OSCC cells (Figure 1F). Furthermore, Nala treatment following LDHA knockdown markedly promoted the proliferation, migration, and invasion of OSCC cells (Figure 1G-J). Taken together with previous study^15^, these results indicate that LDHA undergoes high levels of Kla in OSCC, and the oncogenic capacity of LDHA may be exerted through regulating global cellular Kla abundance, and elevated LDHA lactylation serves as one potential mechanism facilitating OSCC progression.

### Lactylation at LDHA-K318 promotes the malignant progression of OSCC

The secondary mass spectra from LC-MS/MS analysis revealed that lysine 81 (K81) and lysine 318 (K318) were potential Kla sites of LDHA (Figure 2A). To determine the principal Kla site, we first generated OSCC cell lines with stable knockdown of endogenous LDHA, which were subsequently reconstituted with wild-type LDHA (HA-LDHA-WT, referred to as WT) and site-specific mutants (HA-LDHA-K81R and HA-LDHA-K318R, referred to as K81R and K318R) (Figure 2B). The results demonstrated that mutation of K318 to K318R resulted in a significant reduction in LDHA-Kla levels, whereas mutation of K81 to K81R had no notable impact in SCC25 and HSC3 cells (Figure 2B). Furthermore, simultaneous treatment with Nala and 2DG resulted in significant alterations in LDHA-Kla levels in WT groups, whereas K318R groups exhibited low levels of LDHA-Kla with no obvious changes (Figure 2D-E). These findings confirm that K318 is the major Kla site of LDHA. Additionally, CCK-8 and colony formation assays validated that Kla of LDHA-K318 (LDHA-K318la) markedly enhances the proliferative capacity of OSCC cells (Figure 2F-G). Moreover, Transwell assays indicated that LDHA-K318la promotes cell migration and invasion (Figure 2H). Treatment with Nala produced no significant changes in proliferation, migration and metastasis, that may be attributed to the saturated Kla level of LDHA-K318 (Figure 2F-H). However, the oncogenic phenotype driven by LDHA-K318 lactylation was substantially suppressed upon 2-DG addition (Figure 2F-H). Taken together, these findings established that K318 served as the primary Kla site on LDHA, and LDHA-K318la underscored its role in driving malignant progression in OSCC.

### GCN5 plays role as the lactyltransferase of LDHA-K318 in OSCC

To date, several lactyltransferases have been identified, including multiple acetyltransferases and AARS1. To investigate the lactyltransferase responsible for regulating LDHA-Kla in OSCC, WT cells were treated with Nala for 24h, followed by Co-IP assays. The results demonstrated an increased interaction between GCN5 and LDHA, whereas no significant changes were observed in the binding of CBP, P300, AARS1, PCAF, MOF, or TIP60 to LDHA (Figure 3A). IF results indicated that GCN5 can modulate LDHA-Kla levels (Figure 3B). Furthermore, treatment of WT cells with MB-3, a specific small-molecule inhibitor of GCN5, significantly reduced the lactylation level of LDHA and diminished the interaction between GCN5 and LDHA (Figure 3C). Moreover, knockdown of GCN5 attenuated the co-immunoprecipitation of GCN5 with LDHA and concomitantly diminished LDHA-Kla levels (Figure S1A-B, Figure 3D). These findings indicate that GCN5 mediates LDHA-Kla in OSCC.

We first assessed GCN5 expression in OSCC. TCGA database analysis reveals that GCN5 is highly expressed in various tumor types (Figure S2A). Compared with adjacent normal tissues, GCN5 expression is significantly upregulated in both paired and unpaired OSCC tissues (Figure S2B-C). Further analysis demonstrated that the expression level of GCN5 in OSCC was closely associated with pathological differentiation (Grade), T classification, and clinical stage (Figure S2D-F). Moreover, validation using clinical specimens and OSCC cell lines confirmed elevated GCN5 expression in OSCC tissues and cells, and the expression level of GCN5 closely correlated with T classification, N classification, and clinical stage in OSCC patients (Tumor=71, Adjacent=20) (Figure 3E-G). Functionally, pharmacological inhibition of GCN5 with MB-3 suppressed proliferation, migration, and invasion in both WT and K318R groups, with the K318R group displaying heightened sensitivity (Figure 3H-J). To evaluate the oncogenic role of LDHA-Kla *in vivo*, we subcutaneously implanted OSCC cells with stable LDHA knockdown and complemented them with exogenous overexpression of LDHA-WT (WT group) or LDHA-K318R (K318R group) into NOD/SCID mice (Figure 3K). Consistent with *in vitro* observations, LDHA-Kla deficiency significantly attenuated tumor growth *in vivo* (Figure 3L-N). IHC staining revealed notably stronger Ki67 and Pan-Kla signals in the WT group compared to the K318R group (Figure 3O). MB-3 treatment markedly suppressed tumor growth in both the WT and K318R groups (Figure 3L-N). Ki67 staining results indicated that the proliferative capacity was significantly reduced in the K318R+NC, K318R+MB-3, and WT+MB-3 groups compared to the WT+NC group, with the most pronounced proliferation inhibition observed in the K318R+MB-3 group (Figure 3O). Furthermore, Pan-Kla staining of OSCC xenografts in the four groups showed that the staining intensity in the WT+NC group was higher than that in the other three groups (Figure 3O). Collectively, we reveal that GCN5 is upregulated in OSCC and functions as an upstream lactyltransferase for LDHA, inducing lactylation of K318 to promote OSCC progression via enhancing cell proliferation, migration, and invasion. GCN5 inhibition suppresses the oncogenic effects induced by LDHA-K318 lactylation.

### HDAC1 is the potential eraser of LDHA lactylation in OSCC

HDAC1/2/3 and SIRT1/2/3 have been recognized as key deacetylases (erasers) for lysine lactylation. To screen for enzymes potentially involved in the delactylation of LDHA-Kla, OSCC cells transfected with LDHA were treated with the deacetylase inhibitor Panobinostat and Nicotinamide (NAM), respectively. IP assays revealed that NAM did not appreciably alter LDHA-Kla levels, whereas Panobinostat markedly increased them, suggesting that HDAC1/2/3, rather than SIRT1/2/3, served as potential erasers for LDHA-Kla (Figure 4A). Furthermore, treatment with Panobinostat in both WT and K318R cells again confirmed that it upregulates LDHA-Kla levels specifically in WT cells (Figure 4B). Importantly, Panobinostat and NAM treatment did not affect the protein expression of HDAC1/2/3 or LDHA, indicating that these treatments modulates LDHA-Kla by inhibiting HDAC enzymatic activity (Figure 4C). To further confirm the specific enzyme responsible for LDHA delactylation, we performed a targeted screen by silencing HDAC1, HDAC2, or HDAC3 using siRNA (Figure 4D). Knockdown of HDAC1, but not HDAC2 or HDAC3, significantly elevated LDHA-Kla levels (Figure 4E). Knockdown of HDAC1 significantly reduced the interaction between HDAC1 and LDHA (Figure 4F). In conclusion, these results establish HDAC1 as the primary eraser governing LDHA-Kla in OSCC.

### LDHA-K318 lactylation does not affect LDHA protein degradation or nucleocytoplasmic localization in OSCC

Various PTMs of LDHA have been reported to modulate its protein stability, subcellular localization, and enzymatic activity. We first investigated the effect of LDHA-Kla on intracellular localization of LDHA in OSCC cells. OSCC cells were treated with escalating concentrations of Nala or 2DG, followed by nuclear and cytoplasmic fractionation. WB revealed that LDHA was predominantly distributed in the cytoplasm, and neither Nala nor 2DG altered the cytoplasmic abundance of LDHA (Figure 5A). Similarly, in cells expressing either LDHA-WT or LDHA-K318R, supplementation with Nala or 2DG did not provoke any detectable relocalization of LDHA between the nucleus and cytoplasm (Figure 5B). Furthermore, IF staining confirmed that alterations in LDHA-Kla levels driven by either endogenous factors (LDHA-WT vs. LDHA-K318R) or exogenous interventions (Nala and 2DG) exerted no effect on the predominant cytoplasmic distribution of LDHA (Figure 5C).

Subsequently, we focused on exploring the impact of LDHA-Kla on the degradation of LDHA protein. OSCC cells were treated with Nala and 2DG for 0, 6,12 and 24 hours, followed by assessment of LDHA protein levels, which revealed no significant alterations (Figure 5D). Similarly, modulating LDHA-Kla levels via either endogenous manipulation (WT vs. K318R) or exogenous intervention (Nala and 2DG) did not elicit obvious changes in LDHA protein abundance (Figure 5E). CHX chase assays were then conducted to inhibit protein synthesis in cells stably expressing LDHA-WT or LDHA-K318R. WB analysis revealed that LDHA protein levels were significantly decreased due to pronounced degradation at 12 hours in both the WT and K318R groups (Figure 5F). These results indicated that alterations in LDHA-Kla levels do not affect LDHA protein degradation.

### Lactylation of LDHA-K318 enhances its enzymatic activity in OSCC

Finally, we investigated the effect of LDHA-Kla on LDHA enzymatic activity. Previous studies have confirmed that exogenous manipulation of lactylation substrates can markedly alter global Kla levels in OSCC cells^15^. Intracellular lactate content was measured in OSCC cells treated with Nala and 2DG, respectively. The results demonstrated that Nala treatment significantly increased intracellular lactate levels, whereas 2DG treatment exerted the opposite effect (Figure 6A). Meanwhile, mutation of the lactylation site (K318R) markedly reduced Kla levels in OSCC cells and decreased intracellular lactate content (Figure 6B-C). Based on these findings, LDHA enzymatic activity was assessed after purification of LDHA protein from cells expressing LDHA-WT and LDHA-K318R. Notably, only the LDHA-K318R group exhibited a significant reduction in enzymatic activity (Figure 6D). Furthermore, both *in vitro* and *in vivo* experiments revealed that MB-3, the GCN5 inhibitor, significantly suppressed intracellular lactate production and Kla levels (Figure 3M, 6E). After culturing for 24 h with MB-3, LDHA enzymatic activity was evidently inhibited, accompanied by decreased lactate generation (Figure 6F-G). In summary, elevated LDHA-Kla in OSCC promotes lactate production by upregulating LDHA enzymatic activity, thereby contributing to the malignant progression of OSCC.

### Inhibiting LDHA-K318 lactylation with a peptidic inhibitor impedes the malignant progression of OSCC

Previous studies have validated that peptides targeting lactylation sites can markedly decrease protein lactylation levels^33^. Drawing on this approach, we designed a short peptide targeting the LDHA-K318 lactylation site, designated K318-peptide (K318-pe) (Figure 7A). As illustrated in Figure 7B, K318-pe exerted a pronounced inhibitory effect on Kla levels of LDHA-WT rather than LDHA-K318R. Furthermore, the combined treatment with MB-3 and K318-pe resulted in stronger suppression of LDHA-Kla (Figure 7C). Functional assays further revealed that K318-pe attenuated OSCC cell proliferation, migration, and invasion by downregulating LDHA-Kla levels (Figure 7D-F). Notably, the combination of K318-pe and MB-3 led to enhanced suppression of the malignant behaviors of OSCC cells (Figure 7D-F). To further substantiate the antitumor efficacy of K318-pe and MB-3 *in vivo*, PDX models of OSCC were established in this study (Figure 7G). As shown in Figures 7H-J, both K318-pe and MB-3 obviously suppressed tumor growth, and their combined administration yielded a more potent antitumor response. Additionally, no significant visceral toxicity was observed following treatment with K318-pe or MB-3 in mice, as assessed by histopathological analysis of the heart, liver, lung, and kidney (Figure 7K) (Figure 7K). Moreover, treatment with K318-pe or MB-3 alone, as well as their combination, substantially lowered the staining intensities of Kla and Ki67 in OSCC tumor tissues (Figure 7L). Together, these findings indicate that the K318-pe, via specifically targeting LDHA-K318, markedly abrogates the pro-tumorigenic effects driven by hyperlactylation of LDHA-K318. Notably, co-administration of K318-pe with MB-3 achieves synergistic anti-tumor efficacy in OSCC, underscoring its promise as a novel therapeutic intervention.

## Discussion

Numerous studies have linked various PTMs to the initiation and progression of OSCC^34^-^37^. The malignant transformation of oral epithelial cells, along with tumor progression and lymph node metastasis, is associated with extensive alterations in membrane protein glycosylation^38^-^40^. Inhibition of lysine acetylation (Kac) is thought to impair ribosomal function and protein synthesis, potentially contributing to OSCC pathogenesis^41^. Moreover, Kac may influence tumor malignancy by modulating ferroptosis in OSCC^41^. Collectively, these findings support that targeting PTMs represents a promising therapeutic strategy for OSCC. In 2019, the discovery of Kla opened a new avenue for understanding how glycolysis and lactate regulate tumor progression, marking a significant advance in the study of PTM-mediated tumor biology^24^. Since then, researchers have verified that histone and non-histone lactylation modulates the malignant pathological processes of various tumors, including gastric cancer, melanoma, prostate cancer, renal cancer, and liver cancer^12^, ^27^, ^42^-^46^. Our preliminary findings revealed elevated lactate levels in OSCC tissues and cells, which promoted extensive protein lactylation. We mapped the OSCC protein lactylation profile via LC-MS/MS, and bioinformatics analysis identified significant enrichment of multiple metabolic enzymes in the glycolytic pathway, including lactate dehydrogenase (LDH)^15^. These results imply that lactylation participates in regulating metabolic reprogramming in OSCC.

LDH occurs as homotetramers or heterotetramers composed of varying ratios of LDHA and LDHB subunits^18^, ^47^. LDHA has been shown to regulate global lactylation levels by modulating intracellular lactate concentrations^48^-^50^. In the present study, we fully confirmed that LDHA exhibits a high Kla level in OSCC tissues and cells, and the Kla level of LDHA is significantly positively affected by intracellular lactate concentration. Knockdown of LDHA significantly suppressed OSCC proliferation, migration, and invasion, whereas lactate supplementation reversed the proliferative inhibition induced by LDHA deficiency, suggesting that the oncogenic role of LDHA may be achieved by regulating the cellular lactylation process (Figure 7M). These findings are consistent with observations reported by Li *et al*.^51^.

LDHA, as a key driver of tumorigenesis, is subject to regulation by diverse PTMs^20^-^23^. LDHA-K5 acetylation in pancreatic cancer dampens its activity and promotes its lysosomal degradation^21^. LDHA-Y10 phosphorylation in breast cancer activates LDHA, which supports invasion and metastasis by reinforcing redox homeostasis to avert anoikis^22^. Succinylation at K222 in gastric cancer prevents lysosomal degradation of LDHA, thereby enhancing metastatic potential^20^. Methylation at R112 elevates LDHA activity and glycolytic output, fueling tumor cell proliferation^23^. In this work, K318 on LDHA was resolved as the dominant lactylation residue in OSCC. Mutation of K318 curtailed lactate synthesis and diminished global protein lactylation, paralleled by a marked impairment of malignant potential in OSCC both *in vivo* and *in vitro*. Mechanistically, LDHA-K318 lactylation spared the nucleocytoplasmic distribution and protein degradation of LDHA, instead exerting its effect via enzymatic activity to govern lactate output. Together, these findings argue for a positive autoregulatory circuit in which lactylation reinforces LDHA activity, stimulating lactate synthesis that subsequently fuels global lactylation. This mechanism positions that elevated tumor-derived lactate levels may amplify the Warburg effect through lactylation-mediated enhancement of LDHA activity (Figure 7L).

In mammals, three major acetyltransferase families—P300/CREB-binding protein, MOF, and GCN5-related N-acetyltransferases—participate in regulating lactylation^10^, ^24^, ^45^, ^52^, ^53^. Beyond these classical acetyltransferases, alanyl-tRNA synthetase 1 (AARS1) has recently been uncovered as the crucial lactyltransferase^29^-^31^. Drawing on these insights, we set out to pinpoint specific and efficient lactyltransferases responsible for LDHA-Kla at K318. Based on our results, GCN5 emerged as the critical writer for LDHA-Kla. In OSCC, GCN5 was highly expressed, with its levels linked to poor prognostic indicators. Notably, treatment with MB-3, a small-molecule GCN5 inhibitor, markedly suppressed LDHA lactylation, and GCN5 inhibition effectively blocked the protumor effects conferred by LDHA-K318 lactylation.

Considerable efforts have been invested in small-molecule and gene-based strategies targeting lactate production within the biological studies of lactylation^54^-^56^. At present, strategies aimed at modulating lactylation are largely built around lactyltransferase inhibitors. In a recent advance, Chen *et al*. proposed an alternative approach based on peptides engineered to recognize specific lactylation residues^33^. Their work showed that a peptide targeting MRE11 lactylation at K673 (K673-pe) impairs homologous recombination and heightens the sensitivity of cancer cells to cisplatin and PARP inhibitors^33^. With LDHA-K318 lactylation established as a positive driver of malignant progression in OSCC, we generated PDX mouse models and administered two agents aimed at disrupting LDHA-Kla: the lactyltransferase inhibitor MB-3 and K318-pe, a short peptide engineered to abrogate lactylation at K318. Co-administration of MB-3 and K318-pe significantly counteracted the proliferation-promoting effects attributable to LDHA-K318 lactylation. In conjunction with earlier studies, these findings support the concept that precise targeting of specific lactylation sites represents an emerging and promising therapeutic paradigm.

Although multi-layered biochemical, cellular, and PDX *in vivo* assays in the present study have delineated the oncogenic role of LDHA-K318 lactylation in OSCC, several limitations of this work require elaboration. Firstly, there is no commercially available site-specific antibody targeting LDHA-K318 lactylation. This barrier prevents us from performing IHC on clinical OSCC tissues to quantify endogenous LDHA-K318la abundance, as well as conducting systematic correlation analyses between LDHA-K318 lactylation levels and multiple clinicopathological parameters. Additionally, IF cannot be applied to precisely visualize the subcellular co-localization of LDHA-K318 lactylation with GCN5 or HDAC1 within tumor cells. Secondly, rescue experiments with the lactylation-mimetic K318Q mutant at the K318 residue were not included in this work. Supplementary rescue assays using this mutant would provide more direct evidence to strengthen our conclusions. Future work will focus on generating a custom LDHA-K318 lactylation-specific antibody and performing K318Q mimic rescue experiments to further validate the functional role of LDHA-K318 lactylation in OSCC malignant progression. These efforts will facilitate comprehensive tissue validation and translational studies to evaluate its potential as a prognostic biomarker and therapeutic target for OSCC.

## Conclusion

Taken together, our study establishes that LDHA is hyperlactylated in OSCC, with its lactylation levels positively regulated by intracellular lactate concentrations. Lactylation at K318 of LDHA serves to promote OSCC malignancy. Mechanistically, GCN5 and HDAC1 operate as the upstream writer and eraser, coordinating LDHA lactylation to modulate lactate production and thereby drive malignant progression. Combined pharmacological blockade of GCN5 with MB-3 and K318-pe targeting LDHA-K318 lactylation effectively inhibited tumor growth in a PDX model of OSCC. Collectively, these findings uncover a positive feedback loop linking lactate and glycolysis through LDHA lactylation in OSCC, and suggest that interfering with LDHA lactylation may exert broad anti-tumor effects by modulating global protein lactylation. Moreover, this study implicates lactylation-associated regulators as promising therapeutic targets for OSCC, providing insights for clinical development (Figure 7L).

## Supplementary Material

Supplementary figures and tables.

## Figures and Tables

**Figure 1:**
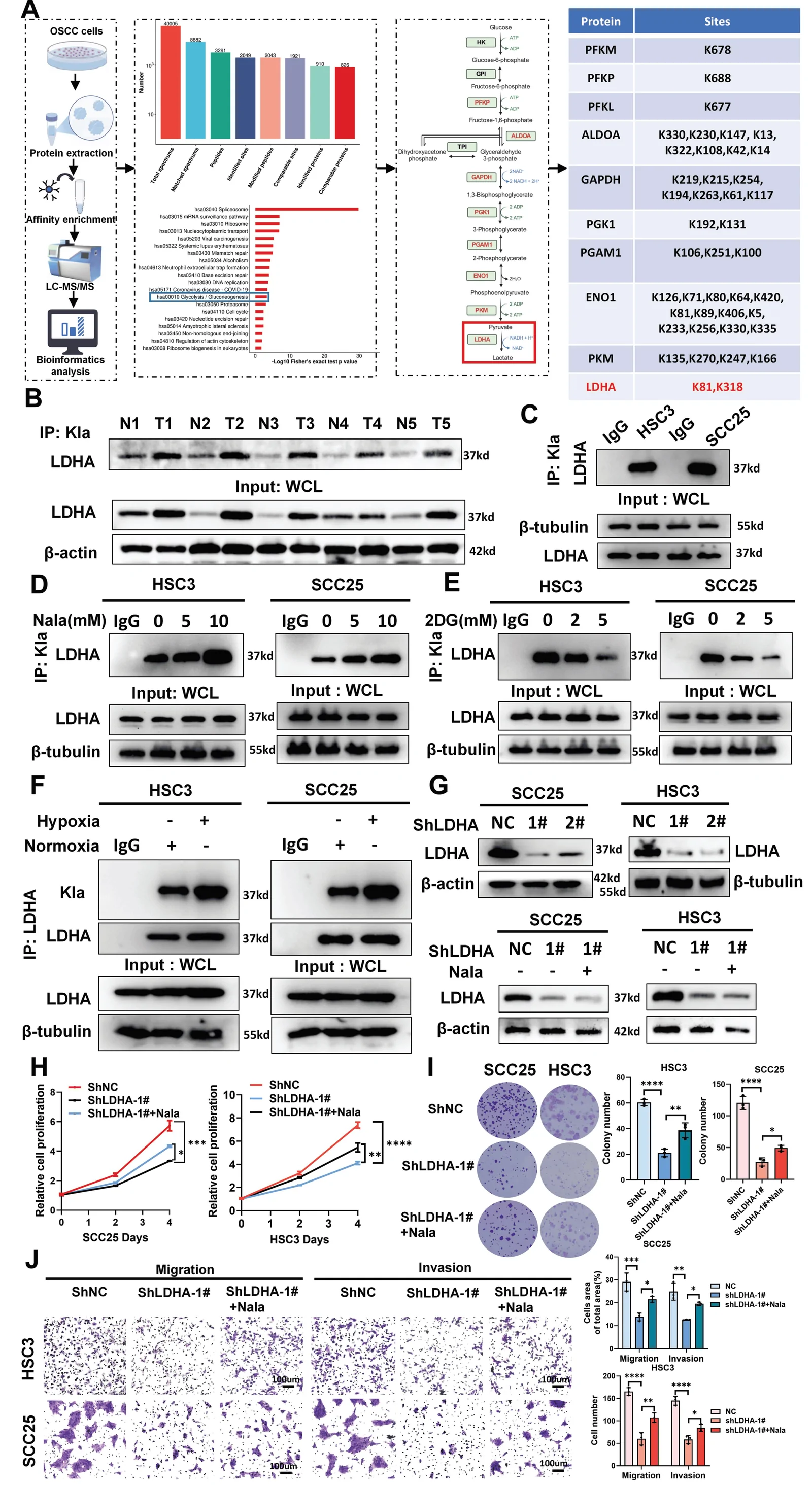
** LDHA is hyperlactylated in OSCC.** (A) LC-MS/MS profiling identified glycolytic pathway components and LDHA as predominant targets of lactylation in OSCC. (B-C) IP coupled with western blotting revealed markedly elevated LDHA-Kla in OSCC tissues (B), as well as in OSCC cell lines (C). (D) Increasing concentrations of Nala (0, 5, 10mM) resulted in enhanced LDHA lactylation in OSCC cells. (E) Glycolytic inhibitor 2DG (0, 2, 5mM) reduced LDHA-Kla levels. (F) Hypoxic conditions upregulated the lactylation status of LDHA. (G) Knockdown efficiency of LDHA was verified, and LDHA expression was examined in LDHA-knockdown OSCC cells cultured with Nala (5mM). (H-J) In LDHA-knockdown cells, Nala (5mM) supplementation restored proliferative capacity (H, I) and significantly enhanced both migration and invasion abilities (J). Scale bar: 100μm. *P<0.05, **P<0.01, ***P<0.001, ****P<0.0001. All the data are presented as mean±SD from three independently performed experiments.

**Figure 2:**
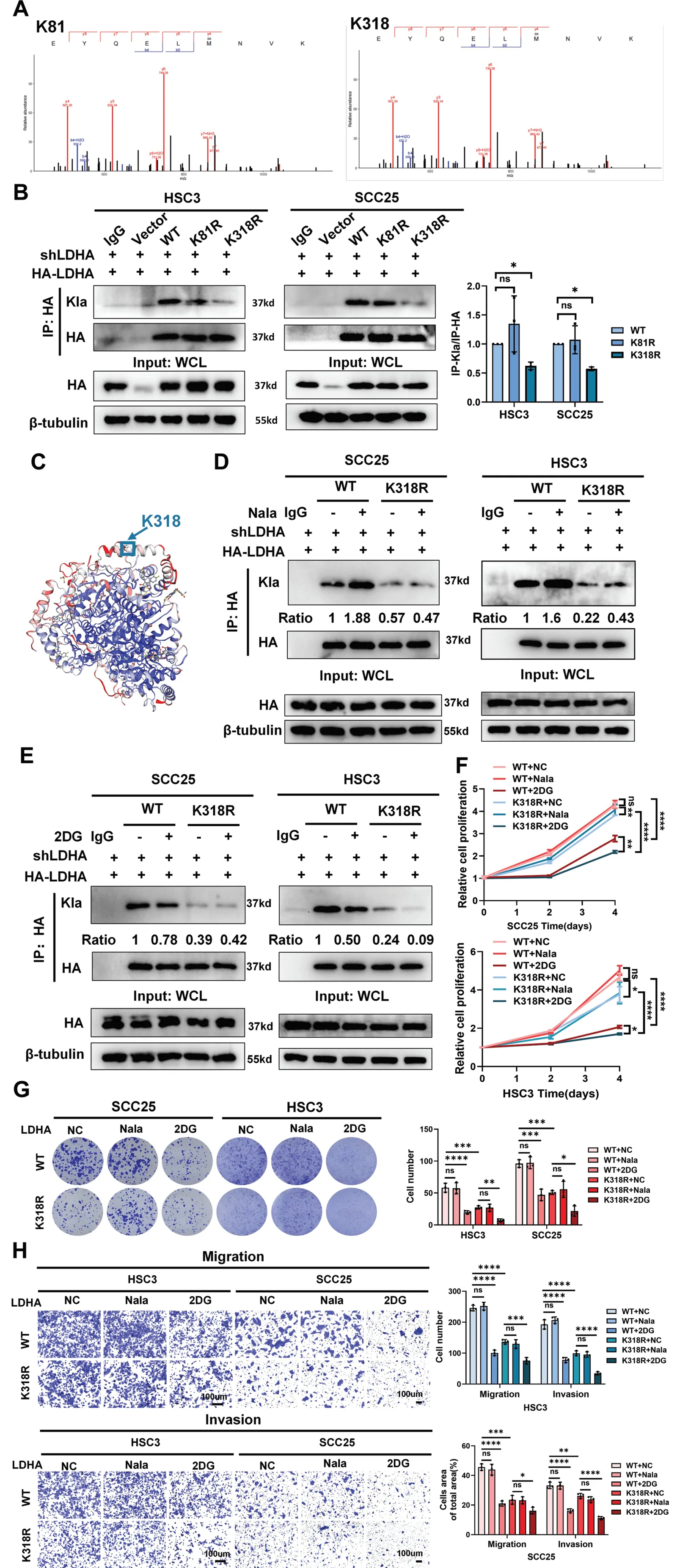
** Lactylation at LDHA-K318 promotes the malignant progression of OSCC.** (A) Potential lactylated peptides derived from LDHA. (B) To validate LDHA-Kla sites in HSC3 and SCC25 cells, mutant strains were constructed using a lentiviral approach. (C) Three-dimensional structure of LDHA constructed by SWISS-MODEL, showing the position of LDHA-K318. (D) The impact of Nala (5mM) on Kla levels was assessed in LDHA-WT and LDHA-K318R cells. (E) The effect of 2DG (2mM) on Kla levels was evaluated in LDHA-WT and LDHA-K318R cells. (F-H) Functional assays demonstrated that LDHA-K318 lactylation promoted OSCC cell proliferation (F-G), as well as migration and invasion (H). Scale bar: 100μm, ns: not significant, *P<0.05, **P<0.01, ***P<0.001, ****P<0.0001. All the data are presented as mean±SD from three independently performed experiments.

**Figure 3:**
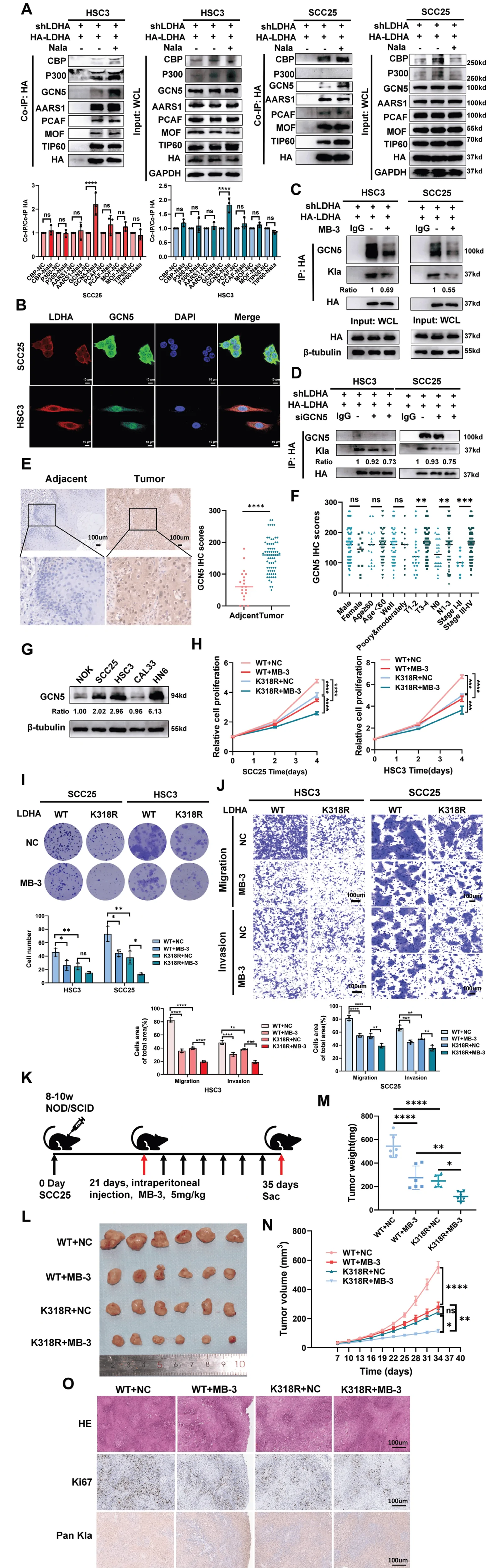
** GCN5 plays role as the lactyltransferase of LDHA-K318 in OSCC.** (A) Co-IP was performed to assess the association of LDHA with various lactyltransferases. Nala:10mM. (B) Colocalization of GCN5 and LDHA in cells. Magnification: ×60, Scale bar: 10μm. (C) The GCN5 inhibitor MB-3 (100uM) markedly decreased the LDHA-Kla levels. (D) Knockdown of GCN5 decreased LDHA-Kla levels. (E) IHC was employed to examine GCN5 expression in clinical OSCC specimens (Tomor=71, Adjacent=20). (F) Clinicopathological correlations between GCN5 and malignant progression in OSCC patients were analyzed. (G) GCN5 was highly expressed in OSCC cell lines. (H-J) Treatment with MB-3 (100μM) modulated the proliferative of LDHA-WT and LDHA-K318R cells (H-I), as well as the migratory and invasive capacity (J) Scale bar: 100μm. (K) Schematic of subcutaneous xenograft model establishment in NOD/SCID mice. (L-N) Effects of LDHA lactylation and GCN5 inhibitor MB-3 on OSCC subcutaneous xenografts. (O) HE and IHC staining for OSCC subcutaneous xenografts. Scale bar: 100μm, ns: not significant, *P<0.05, **P<0.01, ***P<0.001, ****P<0.0001. All the data are presented as mean±SD from three independently performed experiments.

**Figure 4:**
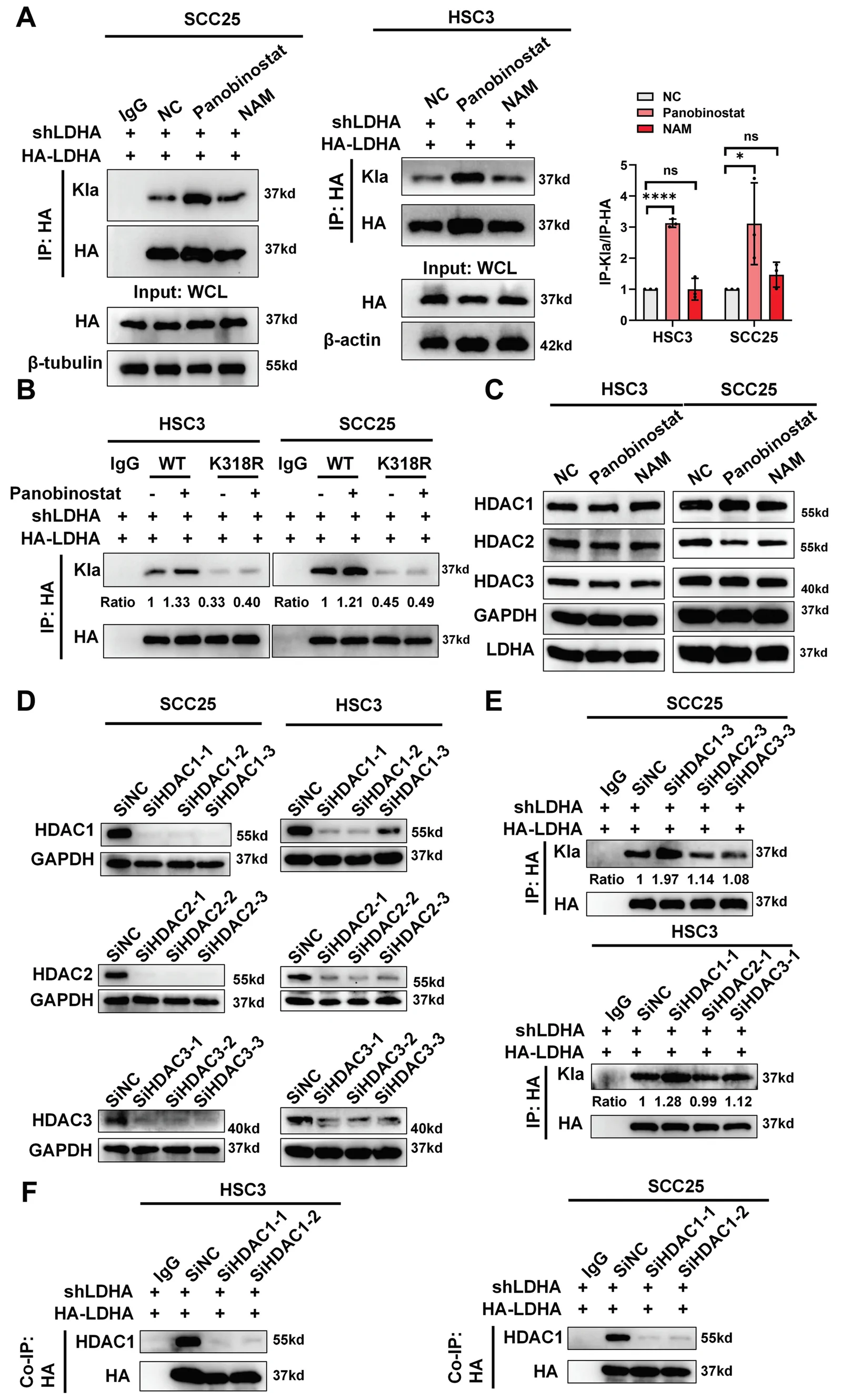
** HDAC1 is the potential eraser of LDHA lactylation in OSCC.** (A) The modulatory effects of Panobinostat (100nM) and nicotinamide (NAM, 10mM) on LDHA-Kla levels were examined. (B) The effect of Panobinostat (100nM) on LDHA-Kla levels in LDHA-WT and LDHA-K318R cells. (C) Neither Panobinostat (100nM) nor NAM (10mM) affected the expression of HDAC1, HDAC2, or HDAC3. (D)Validation of HDAC1/2/3 knockdown efficiency by siHDAC1/2/3 in OSCC cells. (E) Knockdown of HDAC1 exerted a negative regulatory effect on LDHA-Kla levels. (F) Co-IP confirmed the interaction between HDAC1 and LDHA. ns: not significant, *P<0.05, ****P<0.0001. All the data are presented as mean±SD from three independently performed experiments.

**Figure 5:**
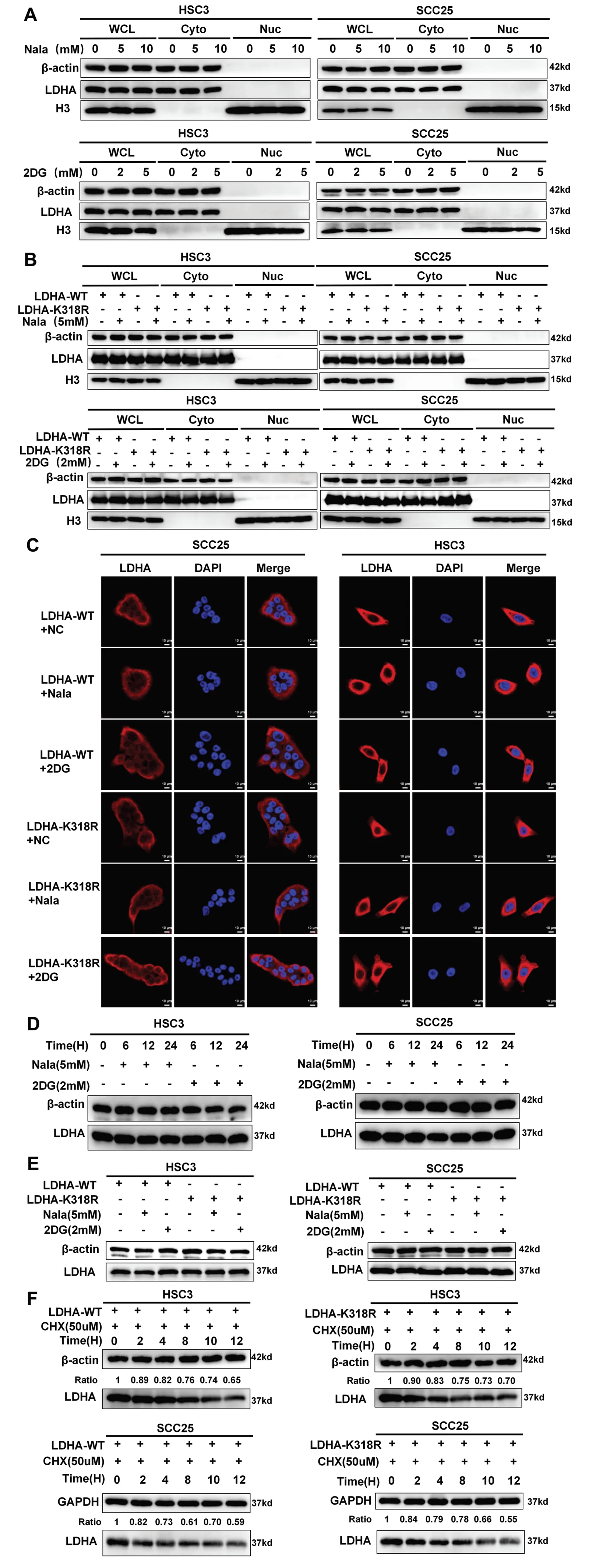
** LDHA-K318 lactylation does not affect LDHA protein degradation or nucleocytoplasmic localization in OSCC.** (A) Alterations in Nala (0, 5, 10 mM) and 2DG (0, 2, 5mM) concentrations were assessed for their impact on the nuclear and cytoplasmic distribution of LDHA protein. (B) The cytoplasmic localization of LDHA was examined in LDHA-WT and LDHA-K318R cells following treatment with Nala (5mM) and 2DG (2mM). (C) IF revealed that LDHA exhibited predominant cytoplasmic localization, with its expression showing no significant correlation with exogenous sodium lactate concentration. (D) The effect of varying treatment durations with Nala and 2DG on LDHA protein expression. (E) Comparable LDHA protein expression levels were observed in LDHA-WT and LDHA-K318R cells following exposure to Nala or 2DG for identical time periods. (F) LDHA-WT and LDHA-K318R cells were treated with CHX (50uM) to monitor LDHA protein expression over time. Magnification: ×60, scale bar: 10μm.

**Figure 6:**
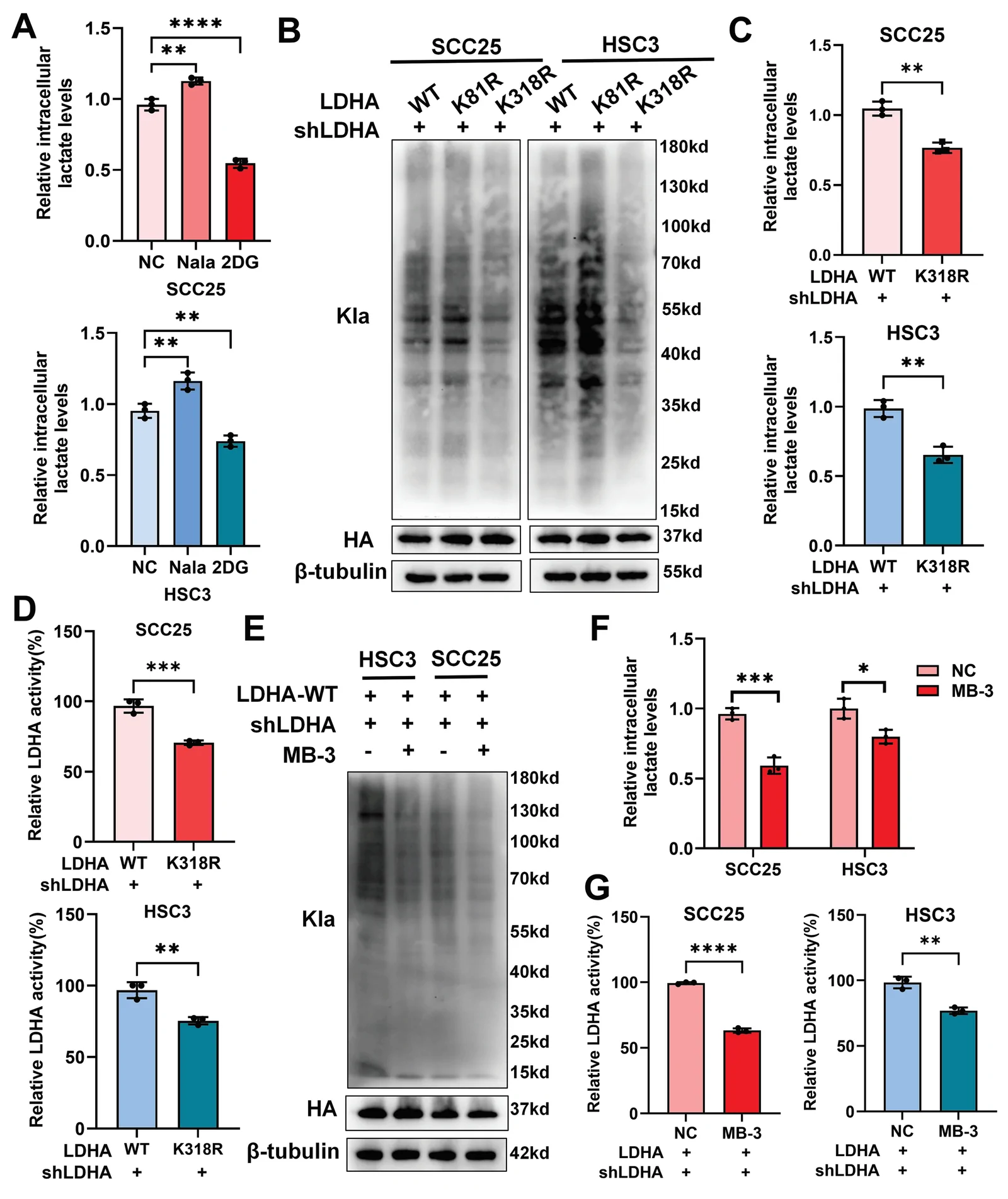
** Lactylation of LDHA-K318 enhances its enzymatic activity in OSCC.** (A) Intracellular lactate levels in OSCC cells were assessed following exogenous supplementation with Nala (5mM) and 2DG (2mM). (B) With mutation of the LDHA-Kla sites, intracellular Kla levels in OSCC cells were evaluated. (C) LDHA-K318R was associated with diminished lactate production within OSCC cells. (D) Introduction of the LDHA-K318R resulted in decreased LDHA enzymatic activity in OSCC cells. (E) Treatment with MB-3 (100uM) modulated intracellular Kla levels. (F) MB-3 (100uM) administration reduced intracellular lactate content. (G) MB-3(100uM) markedly suppressed intracellular LDHA enzymatic activity. *P<0.05, **P<0.01, ***P<0.001, ****P<0.0001. All the data are presented as mean±SD from three independently performed experiments.

**Figure 7:**
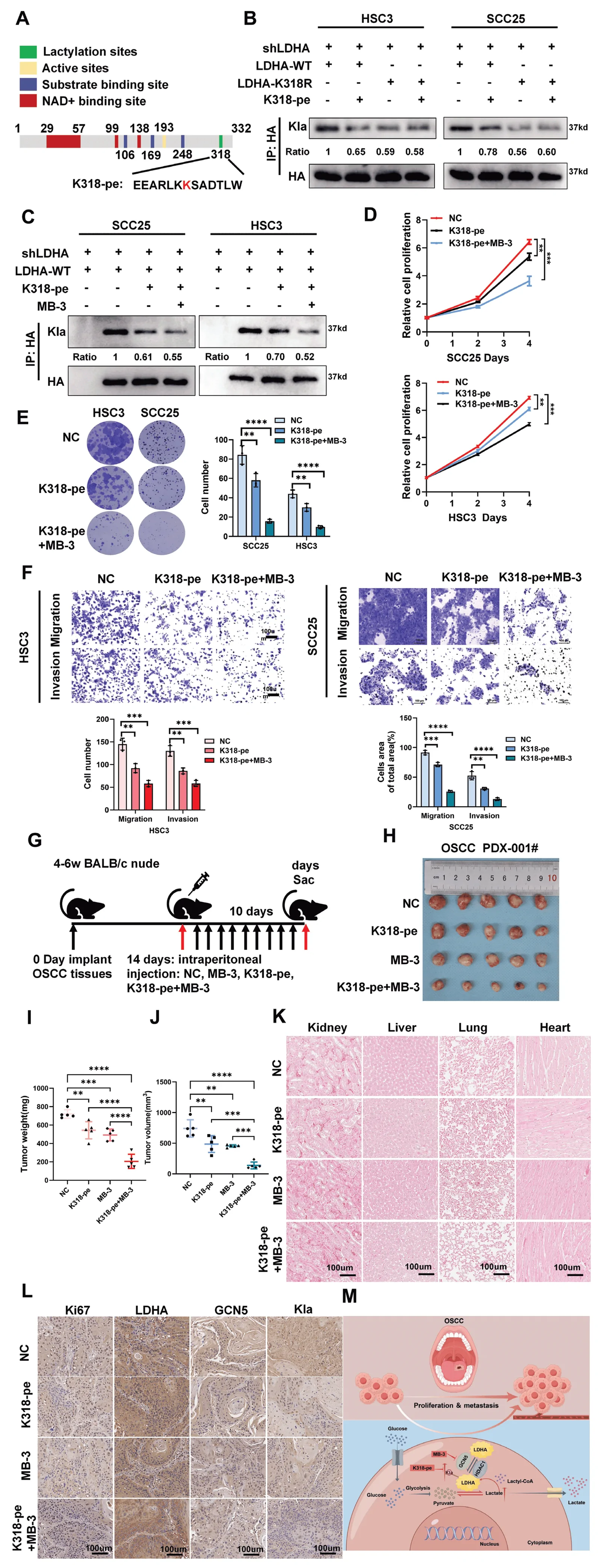
** Inhibiting LDHA-K318 lactylation with a peptidic inhibitor impedes the malignant progression of OSCC.** (A) A short peptide targeting LDHA-K318 was designed. (B) Effects of K318-pe on Kla levels in LDHA-WT and LDHA-K318R. (C) The effects of K318-pe (20μM) and MB-3 (100μM) on LDHA-Kla levels. (D-E) The impact of K318-pe (20μM) and MB-3 (100μM) on OSCC cell proliferation was examined by CCK-8 and colony formation assays. (F) The impact of treatment with K318-pe (20μM) and MB-3 (100μM) on OSCC cell migration and invasion. (G) PDX models of OSCC were established, and mice were allocated to designated treatment groups. (H-J) The effects of K318-peptide (5mg/kg) and MB-3 (5mg/kg) on OSCC tumor growth. (K) Neither K318-pe (5mg/kg) nor MB-3 (5mg/kg) resulted in evident visceral toxicity in mice. (L) Tumor sections from PDX mice were subjected to IHC staining. (M) A graphical model illustrates. GCN5 regulates LDHA Kla, thereby modulating lactate production and promoting OSCC proliferation and metastasis. Scale bar: 100μm, **P<0.01, ***P<0.001, ****P<0.0001. All the data are presented as mean±SD from three independently performed experiments.

## Data Availability

The datasets generated during and/or analyzed during the current study are available from the corresponding author on reasonable request.

## Abbreviations

OSCC: Oral squamous cell carcinoma
Kla: Lysine lactylation
LDHA: Lactate dehydrogenase A
LC-MS/MS: Liquid Chromatography-Tandem Mass Spectrometry
IP: Immunoprecipitation
Co-IP: Co-inmunoprecipitation
WB: Western blot
IF: Immunofluorescence
IHC: Immunohistochemistry
CCK8: Cell counting kit-8
KAT2A: Lysine acetyltransferase 2A
GCN5: General control of amino-acid synthesis 5
PDX: Patient-derived xenograft
MB-3: Butyrolactone 3
AARS1: Alanyl-tRNA synthetase 1
WT: Wild-type
K81R: Mutation of lysine 81 to arginine
K318R: Mutation of lysine 318 to arginine
NAM: Nicotinamide
PTM: Post-translational modification
WCL: Whole cell lysate
Cyto: Cytoplasm
Nuc: Nucleus
CHX: Cycloheximide
