# Supplementary material for: LDHA Lactylation Drives Malignant Progression of Oral Squamous Cell Carcinoma via a Positive Feedback Loop with Lactate
Source: Int J Biol Sci. 2026 Sep 10;22(15):8193–209. doi: 10.7150/ijbs.136949 (PMC13685604; doi:10.7150/ijbs.136949)
Supplement: Supplementary file 1 [file ijbsv22p8193s1.pdf]

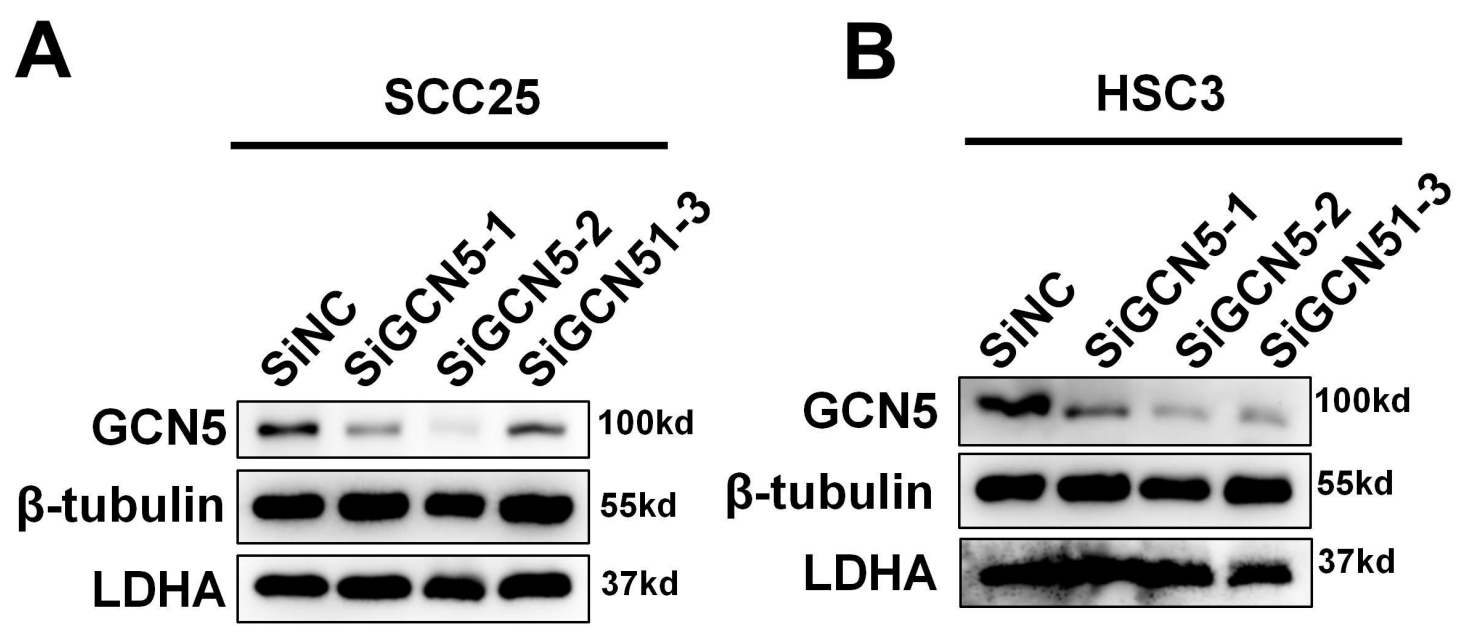

Figure S1 Validation of GCN5 knockdown efficiency. (A) The expression of GCN5 in SCC25. (B) The expression of GCN5 in HSC3.

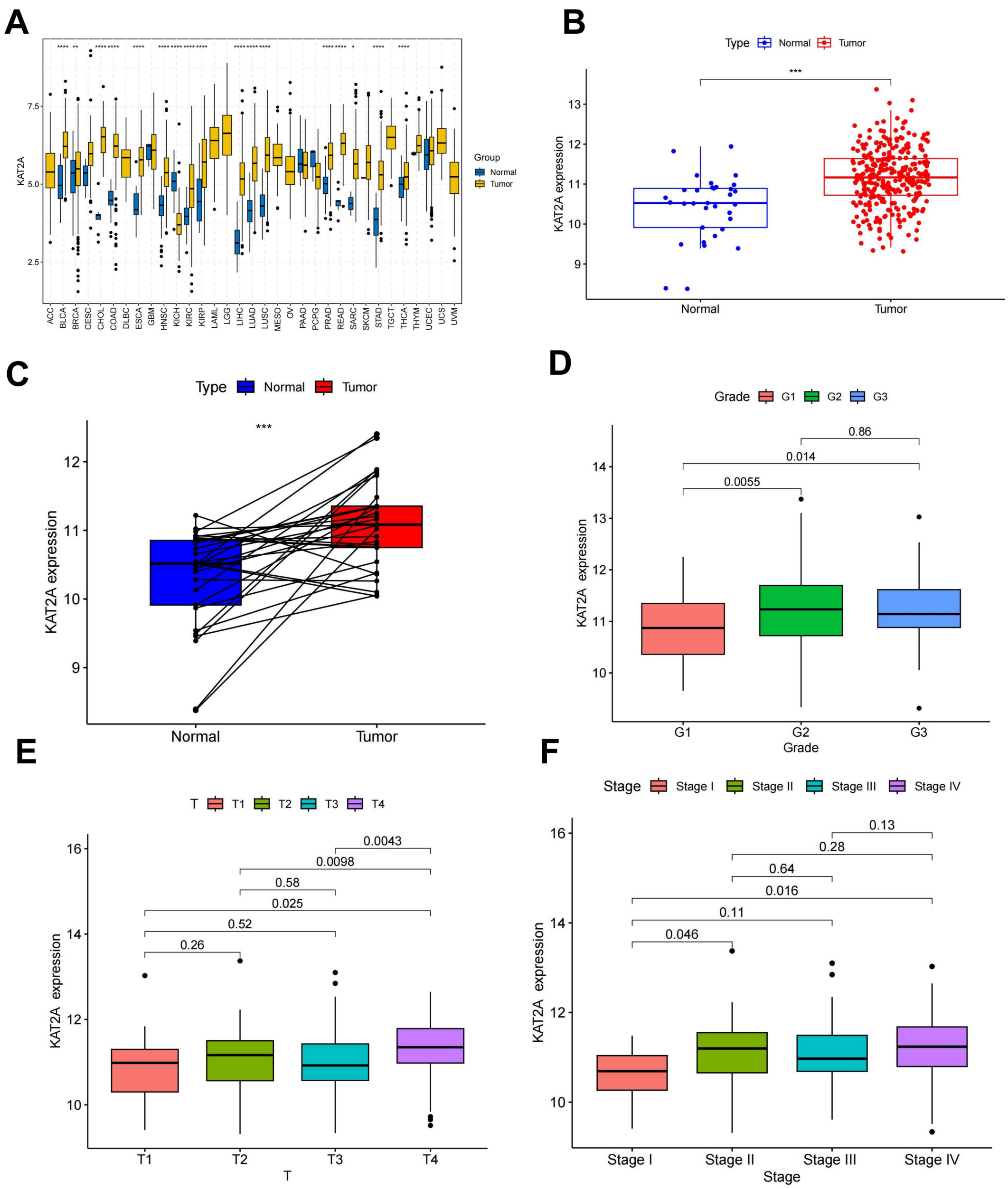

Figure S2 GCN5 exhibits elevated expression in oral squamous cell carcinoma. (A–B) Interrogation of the TCGA dataset reveals upregulated GCN5 expression across multiple tumor types, including OSCC. (C) A comparative analysis of GCN5 expression in OSCC tissues relative to paired adjacent non-cancerous tissues (ANCT). (D-F) High expression of GCN5 is positively correlated with poor prognostic indicators of OSCC, including pathological differentiation (D), T stage (E), and clinical stage (F). **P*<0.05, ***P*<0.01, ****P*<0.001, *****P*<0.0001.

Table S1 The detailed information on the K318-peptide

Protein name	Protein ID	Sequences	Modification	Molecular Weight	Purity
LDHA	P00338	HLYVSPWGGEE ARLKKSADTLW	N-terminal acetylation, C-terminal amidation	2585.20	> 95%

Table S2 The gene sequence for LDHA shRNA

Gene	Sequences
shLDHA-1	ACCGGTGGAGAAAGCCGTCTTAATTTCAAGAGAAATT AAGACGGCTTTCTCCTTTTTTGAATTC
shLDHA-2	ACCGGTGGCAAAGACTATAATGTAATTCAAGAGATTA CATTATAGTCTTTGCCTTTTTTGAATTC

Table S3 The gene sequence for LDHA-overexpressing

Gene	Sequences
LDHA-WT	ATGGCAACTCTAAAGGATCAGCTGATTTATAATCTTCTAAAGGAAGAA CAGACCCCCCAGAATAAGATTACAGTTGTTGGGGTTGGTGCTGTT GGCATGGCCTGTGCCATCAGTATCTTAATGAAGGACTTGGCAGATG AACTTGCTCTTGTTGATGTCATCGAAGACAAATTGAAGGGAGAGA TGATGGATCTCCAACATGGCAGCCTTTTCCTTAGAACACCAAAGAT TGTCTCTGGCAAAGACTATAATGTAACTGCAAACTCCAAGCTGGT CATTATCACGGCTGGGGCACGTCAGCAAGAGGGCGAGAGTAGGC TGAATTTGGTCCAGCGTAACGTGAACATCTTTAAATTCATCATTCCT AATGTTGTAAAATACAGCCCGAACTGCAAGTTGCTTATTGTTTCAA ATCCAGTGGATATCTTGACCTACGTGGCTTGGAAGATAAGTGGTTT TCCCAAAAAACCGTGTTATTGGAAGCGGTTGCAATCTGGATTCAGC CCGATTCCGTTACCTAATGGGGGAAAGGCTGGGAGTTCACCCATT

	AAGCTGTCATGGGTGGGTCCTTGGGGAACATGGAGATTCCAGTGT<br>GCCTGTATGGAGTGGAATGAATGTTGCTGGTGTCTCTCTGAAGAC<br>TCTGCACCCAGATTTAGGGACTGATAAAGATAAGGAACAGTGGAA<br>AGAGGTTCACAAGCAGGTGGTTGAGAGTGCTTATGAGGTGATCAA<br>ACTCAAAGGCTACACATCCTGGGCTATTGGACTCTCTGTAGCAGAT<br>TTGGCAGAGAGTATAATGAAGAATCTTAGGCGGGTGCACCCAGTT<br>TCCACCATGATTAAGGGTCTTTACGGAATAAAGGATGATGTCTTCC<br>TTAGTGTTCCTTGCATTTTGGGACAGAATGGAATCTCAGACCTTGT<br>GAAGGTGACTCTGACTTCTGAGGAAGAGGCCCGTTTGAAGAAGA<br>GTGCAGATACACTTTGGGGGATCCAAAAGGAGCTGCAATTTTACC<br>CATACGATGTTCCAGATTACGCTTAAGCGGCCGC
LDHA-K81R	ATGGCAACTCTAAAGGATCAGCTGATTTATAATCTTCTAAAGGAAGAA<br>CAGACCCCCCAGAATAAGATTACAGTTGTTGGGGTTGGTGCTGTT<br>GGCATGGCCTGTGCCATCAGTATCTTAATGAAGGACTTGGCAGATG<br>AACTTGCTCTTGTTGATGTCATCGAAGACAAATTGAAGGGAGAGA<br>TGATGGATCTCCAACATGGCAGCCTTTTCCTTAGAACACCAAAGAT<br>TGTCTCTGGCAGAGACTATAATGTAACTGCAAACTCCAAGCTGGT<br>CATTATCACGGCTGGGGCACGTCAGCAAGAGGGCGAGAGTAGGC<br>TGAATTTGGTCCAGCGTAACGTGAACATCTTTAAATTCATCATTCCT<br>AATGTTGTAAAATACAGCCCGAACTGCAAGTTGCTTATTGTTTCAA<br>ATCCAGTGGATATCTTGACCTACGTGGCTTGGAAGATAAGTGGTTT<br>TCCCAAAAACCGTGTTATTGGAAGCGGTTGCAATCTGGATTCAGC<br>CCGATTCCGTTACCTAATGGGGGAAAGGCTGGGAGTTCACCCATT<br>AAGCTGTCATGGGTGGGTCCTTGGGGAACATGGAGATTCCAGTGT<br>GCCTGTATGGAGTGGAATGAATGTTGCTGGTGTCTCTCTGAAGAC<br>TCTGCACCCAGATTTAGGGACTGATAAAGATAAGGAACAGTGGAA<br>AGAGGTTCACAAGCAGGTGGTTGAGAGTGCTTATGAGGTGATCAA<br>ACTCAAAGGCTACACATCCTGGGCTATTGGACTCTCTGTAGCAGAT<br>TTGGCAGAGAGTATAATGAAGAATCTTAGGCGGGTGCACCCAGTT

	TCCACCATGATTAAGGGTCTTTACGGAATAAAGGATGATGTCTTCC<br>TTAGTGTTCCTTGCATTTTGGGACAGAATGGAATCTCAGACCTTGT<br>GAAGGTGACTCTGACTTCTGAGGAAGAGGCCCGTTTGAAGAAGA<br>GTGCAGATACACTTTGGGGGATCCAAAAGGAGCTGCAATTTTACC<br>CATACGATGTTCCAGATTACGCTTAAGCGGCCGC
LDHA-K318R	ATGGCAACTCTAAAGGATCAGCTGATTTATAATCTTCTAAAGGAAGAA<br>CAGACCCCCCAGAATAAGATTACAGTTGTTGGGGTTGGTGCTGTT<br>GGCATGGCCTGTGCCATCAGTATCTTAATGAAGGACTTGGCAGATG<br>AACTTGCTCTTGTTGATGTCATCGAAGACAAATTGAAGGGAGAGA<br>TGATGGATCTCCAACATGGCAGCCTTTTCCTTAGAACACCAAAGAT<br>TGTCTCTGGCAAAGACTATAATGTAACTGCAAACTCCAAGCTGGT<br>CATTATCACGGCTGGGGCACGTCAGCAAGAGGGCGAGAGTAGGC<br>TGAATTTGGTCCAGCGTAACGTGAACATCTTTAAATTCATCATTCCT<br>AATGTTGTAAAATACAGCCCGAACTGCAAGTTGCTTATTGTTTCAA<br>ATCCAGTGGATATCTTGACCTACGTGGCTTGGAAGATAAGTGGTTT<br>TCCCAAAAACCGTGTTATTGGAAGCGGTTGCAATCTGGATTCAGC<br>CCGATTCCGTTACCTAATGGGGGAAAGGCTGGGAGTTCACCCATT<br>AAGCTGTCATGGGTGGGTCCTTGGGGAACATGGAGATTCCAGTGT<br>GCCTGTATGGAGTGGAATGAATGTTGCTGGTGTCTCTCTGAAGAC<br>TCTGCACCCAGATTTAGGGACTGATAAAGATAAGGAACAGTGGAA<br>AGAGGTTCACAAGCAGGTGGTTGAGAGTGCTTATGAGGTGATCAA<br>ACTCAAAGGCTACACATCCTGGGCTATTGGACTCTCTGTAGCAGAT<br>TTGGCAGAGAGTATAATGAAGAATCTTAGGCGGGTGCACCCAGTT<br>TCCACCATGATTAAGGGTCTTTACGGAATAAAGGATGATGTCTTCC<br>TTAGTGTTCCTTGCATTTTGGGACAGAATGGAATCTCAGACCTTGT<br>GAAGGTGACTCTGACTTCTGAGGAAGAGGCCCGTTTGAAGAGGA<br>GTGCAGATACACTTTGGGGGATCCAAAAGGAGCTGCAATTTTACC<br>CATACGATGTTCCAGATTACGCTTAAGCGGCCGC

Table S4 The siRNA sequences for HDAC1-3

Genes	Sequences	
	sense（5'-3'）	antisense（5'-3'）
siHDAC1-1#	GCUUCAAUCUAACUAUCAATT	UUGAUAGUUAGAUUGAAGCTT
siHDAC1-2#	GCAAGUGCUGUGAAACUUATT	UAAGUUUCACAGCACUUGCTT
siHDAC1-3#	GCAAGUAUUAUGCUGUUAATT	UUAACAGCAUAAUACUUGCTT
siHDAC2-1#	GGCAAAUACUAUGCUGUCATT	UGACAGCAUAGUAUUUGCCTT
siHDAC2-2#	GCUGGAGCUGUGAAGUUAATT	UUAACUUCACAGCUCCAGCTT
siHDAC2-3#	CGUUAAGGAAGAAGAUAAATT	UUUAUCUUCUUCCUUAACGTT
siHDAC3-1#	CCAAGAGUCUUAAUGCCUUTT	AAGGCAUUAAGACUCUUGGTT
siHDAC3-2#	GCCGCUACUACUGUCUGAATT	UUCAGACAGUAGUAGCGGCTT
siHDAC3-3#	GGAGCUUCCCUAUAGUGAATT	UUCACUAUAGGGAAGCUCCTT

Table S5 The siRNA sequences for GCN5

Genes	Sequences	
	sense（5'-3'）	antisense（5'-3'）
SiGCN5-1#	CCUCGAAUGAGCAGGUCAATT	UUGACCUGCUCAUUCGAGGTT
SiGCN5-2#	GACGAUGUUCGAGCUCUCATT	UGAGAGCUCGAACAUCGUCTT
SiGCN5-3#	GAAGCUGAUUGAGCGCAAATT	UUUGCGCUCAAUCAGCUUCTT

Table S6. Clinical Characteristics and Treatment History of the Chemoresistant OSCC Patient Used for PDX Establishment

Patient ID: OSCC-PDX-001

Clinical Characteristics

Parameter	Details
Age	54
Gender	Male
Smoking History	Yes
Alcohol History	Yes
Primary Diagnosis	Oral squamous cell carcinoma (the upper right gingiva; lower right gingiva; right buccal mucosa)
Tumor Site	Right gingiva (upper & lower) and right bucca
TNM Staging (AJCC 8th ed.)	cT4aN1M0
Clinical Stage	Stage IVA
Histological Grade	Well-differentiated (G1)

Treatment History and Response

Duration	Regimen	Treatment Response (RECIST 1.1)
2024.04 - 2024.06	Chemoimmunotherapy: Nab-Paclitaxel (350mg) + Tislelizumab (200mg)	**Progressive Disease (PD)**
2024.07 - 2024.10	Definitive Chemoradiotherapy:<br>Radiotherapy: GTV 70Gy/33F, CTV1 60Gy/33F, CTV2 54Gy/33F<br>Concurrent Chemotherapy: Cisplatin (60mg)<br>Targeted Therapy: Nimotuzumab	**Progressive Disease (PD)**
2024.10 - 2024.11	Chemoimmunotherapy: Tislelizumab (200mg) + Capecitabine (1.5g)	**Progressive Disease (PD)**
